# A perspective on genetic and polygenic risk scores—advances and limitations and overview of associated tools

**DOI:** 10.1093/bib/bbae240

**Published:** 2024-05-20

**Authors:** Jana Schwarzerova, Martin Hurta, Vojtech Barton, Matej Lexa, Dirk Walther, Valentine Provaznik, Wolfram Weckwerth

**Affiliations:** Department of Biomedical Engineering, Faculty of Electrical Engineering and Communication, Brno University of Technology, Technicka 10, Brno 61600, Czechia; Molecular Systems Biology (MOSYS), Department of Functional and Evolutionary Ecology, University of Vienna, Vienna 1010, Austria; Department of Computer Systems, Faculty of Information Technology, Brno University of Technology, Brno 612 00, Czechia; Department of Biomedical Engineering, Faculty of Electrical Engineering and Communication, Brno University of Technology, Technicka 10, Brno 61600, Czechia; RECETOX, Faculty of Science, Masaryk University, Kotlarska 2, Brno 62500, Czech Republic; Faculty of Informatics, Masaryk University, Botanicka 68a, Brno 60200, Czech Republic; Max-Planck-Institute of Molecular Plant Physiology, Potsdam 14476, Germany; Department of Biomedical Engineering, Faculty of Electrical Engineering and Communication, Brno University of Technology, Technicka 10, Brno 61600, Czechia; Department of Physiology, Faculty of Medicine, Masaryk University, Brno 62500, Czech Republic; Molecular Systems Biology (MOSYS), Department of Functional and Evolutionary Ecology, University of Vienna, Vienna 1010, Austria; Vienna Metabolomics Center (VIME), University of Vienna, Vienna 1010, Austria

**Keywords:** polygenic risk score, genetic variations, GWAS, genomic prediction, genotype, phenotype

## Abstract

Polygenetic Risk Scores are used to evaluate an individual's vulnerability to developing specific diseases or conditions based on their genetic composition, by taking into account numerous genetic variations. This article provides an overview of the concept of Polygenic Risk Scores (PRS). We elucidate the historical advancements of PRS, their advantages and shortcomings in comparison with other predictive methods, and discuss their conceptual limitations in light of the complexity of biological systems. Furthermore, we provide a survey of published tools for computing PRS and associated resources. The various tools and software packages are categorized based on their technical utility for users or prospective developers. Understanding the array of available tools and their limitations is crucial for accurately assessing and predicting disease risks, facilitating early interventions, and guiding personalized healthcare decisions. Additionally, we also identify potential new avenues for future bioinformatic analyzes and advancements related to PRS.

## Introduction

**Figure 1:**
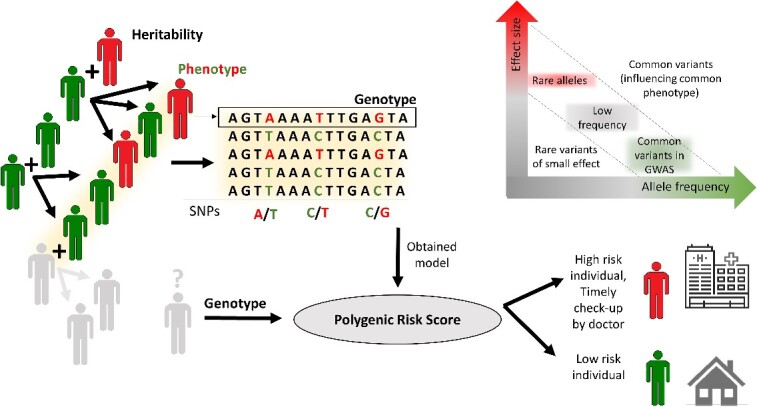
Illustration of the basic concepts of PRS. Individuals of a populations are each characterized by their inherited genotypes and their associated phenotypes. GWAS aims to identify genetic variants that are causaly related with a phenotype of interest. However, it is generally observed that common variants (high minor allele frequency) are associated with only minor phenotypic effects, as large effect genotypic differences are generally selected against, as they are frequently detrimental. Thus, when profiling a general population, large-effect-SNPs are rare. This, and the polygenic origin of many diseases and conditions, necessitates the combination of many markers (SNPs) to arrived at an aggregated risk score, the PRS. This figure is in part adapted from [15, 16].

The concept of Risk Score (RS) calculation has been used in various fields and for many years [1–4]. Risk assessment and related scoring methodologies can be found and have been utilized in various industries such as finance [5], insurance [6], cybersecurity [7] and, of course, healthcare [1] to evaluate and quantify the likelihood and impact of potential risks associated with the parameters and variables of the respective system under study. Scoring systems developed in those areas later inspired genetics and heritability research as well [8], which have become known as calculations of Genetic Risk Score (GRS) [9] or Polygenic Risk Score (PRS) [10]/Polygenic Score (PGS) [11]. Scientists have discussed the polygenic nature of many human phenotypes for some time [12]. However, it was not until relatively recently that Genome-Wide Association Studies (GWAS) [13, 14] provided evidence that the genetic basis of most complex traits largely consists of the cumulative influence of hundreds or even thousands of variants with minor effects [14], and thus, aggregating multiple SNP-loci with minor effect appeared indicated, leading to the concept of PRSs (Fig. 1).

GWAS primarily focuses on identifying associations between individual genetic markers, usually single nucleotide polymorphisms (SNPs), and specific traits or diseases. While GWAS can identify single genetic variants associated with traits, they typically do not directly test for combinations of alleles. Also, identified individual GWAS candidate SNPs are sufficient for diagnostic purposes only if the effect sizes are large, which oftentimes is not the case (Fig. 1). PRS combine the small effects, i.e., the strengths of the association of allelic variants with a particular phenotype, of many individual SNPs (potentially all known SNPs) to result in an aggregated score. As often the study context is with regard to assessing risks for failure/disease, we speak of a ‘risk’ score. The higher the heritability of a trait, the more predictive power PRS is likely to have, as there is a larger proportion of genetic variance that can be captured and used for risk prediction. Conversely, traits with low heritability may be more challenging to predict accurately using PRS alone, as the genetic influence is weaker. PRS, in turn, can be used to investigate the heritability of a particular trait by comparing the proportion of variance explained by the PRS to the total phenotypic variance observed in a population [17]. By assessing the contribution of PRS to the trait’s heritability, we can gain insights into the genetic component of the trait under study. The PRS approach can potentially be expanded to examine the impact of copy number variants, epigenetic markers, and various other factors [18].

PRS calculations have been particularly widely adopted in behavioural genetics for their potential to illuminate the genetic foundations of complex, multigenic behavioural traits. Behavioural genetics [19] focuses on understanding how both genetic and environmental factors contribute to individual differences in behaviour, personality and psychological traits. PRS allows researchers to harness the cumulative effects of numerous genetic variants associated with these behavioural traits. By utilizing PRS, researchers can assess an individuals’ genetic risk-specific behaviours or psychological conditions, such as schizophrenia [20] or depression [21]. This predictive capability offers valuable insights into the interplay between genetic predisposition and the environment, enabling a more comprehensive understanding of the complex nature of behavioural traits. Overall, PRS holds great promise as a powerful tool for investigating the genetic basis of complex behavioural traits and can lead to a deeper understanding of human behaviour and psychological conditions, ultimately contributing to advancements in personalized medicine and behavioural interventions.

The theoretical framework of PRS is closely linked to GWAS. GWAS involves analyzing the entire genome of many individuals to identify genetic variants associated with specific traits or diseases [22]. These studies have been instrumental in uncovering numerous genetic markers linked to a wide range of complex traits and conditions. PRS, on the other hand, leverages the results of GWAS by aggregating the effects of multiple genetic variants associated with a particular trait [23, 24]. This cumulative approach allows researchers to calculate a PRS for an individual, which represents their genetic predisposition to a specific trait or disease based on the presence of these variants. The integration of PRS and GWAS findings has opened up new avenues of research in various fields, including medicine, psychology, and personalized healthcare [25, 26]. PRS can be used to predict an individual’s risk of developing specific diseases or exhibiting certain behavioural traits, aiding in early detection and preventive measures. Additionally, PRS can provide insights into the genetic architecture of complex traits, allowing researchers to identify potential biological pathways and therapeutic targets.

The concept of PRS is a burgeoning field in genetics, still in its early stages of development. As rapid advances in genomics technologies are made, the number of SNPs that can be detected and the population sizes that can be genotyped increase rapidly and significantly [27]. With this rise in genetic data availability, the predictive power of PRSs increases. By incorporating a broader array of genetic variants associated with specific traits or diseases and with larger populations tested for SNP effects, the PRS becomes more robust and accurate in assessing an individual’s genetic risk for complex traits.

Most review articles on PRS present a comprehensive view of different algorithms or point out directly specific challenges [14, 28, 29]. However, a comprehensive overview of the available tools is in need of an update, which we aim to provide with this article.

## Mathematical description

*GRS* [9], or Genetic Index, is the most common approach to evaluate the cumulative effect of many genetic factors with major effects on the phenotype or disease. It can be used to estimate the probability (or risk) for the manifestation of an outcome of interest based on genetic variants.

GRS aggregates effects of allelic variants found in an individual *j* on phenotype by summing over *k* independent genetic variants with a strong association to phenotype based on the determined individual effect size and associated *P*-value [9, 30]:


(1) \begin{equation*} GR{S}_j=\sum_{i=1}^k{\beta}_i{N}_{ij}, \end{equation*}

where ${\beta}_i$ estimates the effect size, expressed as log-odds ratios derived from a logistic regression analysis with additive genetic effects for binary traits or coefficients obtained from linear models for quantitative traits associated with a single allele count, multiplied by the number of respective alleles ${N}_i$ at a given locus *i* in individual *j*.

For example, *N* may take on values of 0, 1, or 2 for a diploid organism, representing, for example, genotypes (‘AA’, ‘aA’, ‘aa’), where A is the reference allele, encoded as ‘0’. Effect sizes, denoted as ${\beta}_i$, are usually derived from GWAS computations. They incorporate adjustments for confounding factors like population structure. The *P*-values generated by GWAS can be utilized to filter for k significant SNPs. However, a crucial limitation arises from the strict additive treatment of variants, which fails to adequately capture interactions—epistasis. Moreover, linear modeling approaches encounter challenges in handling dominant/recessive alleles.

*PRS* is an extension of GRS by including SNP-loci with smaller effect sizes, eventually even all SNPs, regardless of effect size and associated *P*-value [9]. Thus, the difference relative to GRS is only with regard to the chosen *k*, the number of SNP-sites included in the score. Therefore, by including weak associations, the score becomes more ‘*poly-gene-informative*’ than the GRS, and identifies high-risk individuals more precisely [9, 31, 32]. Since PRS requires input from GWAS, computing of PRS is demanding, when the model is first established in a population, but cheap once an individual is genotyped.

The study [9] by Igo Jr. et al. divided PRS calculation approaches into two ways:

(1) *Pruning and Thresholding (P + T)*, also called *Pruning or Clumping*.
This approach addresses linkage disequilibrium, LD, by selecting a representative subset of variants from GWAS to use in the *RS*. Several different procedures for finding significance thresholds related to predicted binary outcomes are as follows [9]:
  a) Selection based on AUC (Area Under the ROC-Receiver Operating Characteristic-Curve) threshold,
  b) pseudo-*R*^2^ [33],
  c) and other parameters of prediction performance.
(2) *Bayesian and Variable reduction models.*
Advanced approaches for calculating of PRS perform regression with correlated data. These approaches calculate with all markers jointly. In general, the Bayesian statistical framework [34] has a prior probability distribution for the parameters of interest and produces an updated posterior distribution given the data. These models utilize summary statistics to estimate shrinkage towards marker effects, taking into account LD information from the reference panel [9, 35–37]. Consequently, a specific distribution is chosen, which significantly contributes to the overall heritability.

### PRS versus Genomic Predictions

Genomic Predictions (GP) is a concept that is closely related to PRS. While GP aims to directly predict a phenotype of interest using whole-genome information, PRS focuses more on aggregating effects of multiple genetic variants associatd with a trait or disease resulting in a score that can be interpreted as a risk to develop a certain phenotpye. The difference is that PRS aggregates individual SNP effect sizes into a global score, whereas GP determines the effect size of SNPs in light of all SNPs simultaneously and depending on the chosen prediction model (e.g., ridge regression).

As is the case for any statistical prediction model, correlated variables pose problems with regard to the stability of parameter estimates and interpretation. For identical (or highly correlated) SNP-patterns as caused by linkage or at different sites in the genome) associated with large effects, classical PRS will add the same effect size twice (unless explicitely accounted for as noted above, or as implemented, for example, by LD score regression (see below)), whereas in GP, by virtue of the typically employed regularized regression method (most promintently Lasso or Rigde regression), only one of the two will be chosen (by Lasso) or both but at reduced effect size estimates (by Ridge regression).

Another differentiating aspect concerns the inclusion of covariates, such as population structure. As the GWAS methodology is generally set up to account for it, for example, via the kinship matrices, GRS/PRS does generally consider population structure as a confounding factor. In GP, this may or may not be done, as often, GP aims at predicting outcome, and not at correctly identifying causal genomic variants.

**Figure 2:**
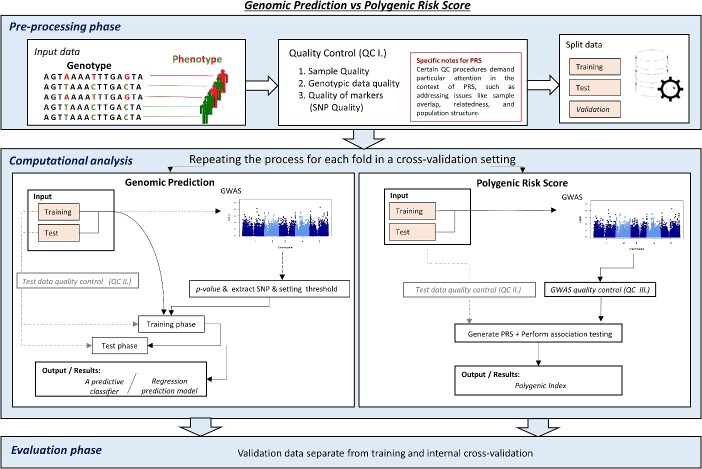
Workflow of procedure for GP and PRS calculations. The pre-processing steps are the same for both methods. GP can include GWAS for extracting SNPs using thresholds, nevertheless, this step is not mandatory for GP. It is also possible to use the whole SNP dataset (although it can be more time consuming for training the models). On the other hand, GWAS is a mandatory step in PRS calculation as it yields the effect sizes associated with every SNP to yield the PRS. In total, there are three Quality Control steps (QC I, QC II and QC III). QC I is connected to checking sequencing data quality, QC II is connected to testing quality control of data for predictive modelling, such as sufficient data variability to avoid overfitting, and finally, QC III, which is connected to the background of the majority available tools. QC III usually uses seven control parts, which are included in PLINK [35] (available on https://zzz.bwh.harvard.edu/plink/) and described in [36].

Given the similarities between PRS and GP, some level of confusion can be noted among researchers. To aid in a better differentiatiation between the two, we present an overview of the standard procedures for calculating both PRS and GP (Fig. 2—workflow of procedure for GP and PRS calulations), which we hope will help highilighting commonalities and differences between PRS and GP.

## Advantages and limitations of the PRS

PRS aims to adequately capture the polygenic nature of many traits by taking into account the collective influence of numerous genetic variants, each with a relatively modest impact. Therefore, PRSs are becoming increasingly popular in the field of genetics and personalized healthcare [37]. PRSs offer personalized risk assessments, aiding individuals in understanding their genetic predisposition to diseases and enabling informed decision-making. Additionally, PRS accelerates genetic research, provides cost-effective genetic risk assessment, and aids in population-level disease risk identification, thus benefiting healthcare and research endeavors.

On the other hand, evidently, the predictive power of PRS is limited by the number of SNPs tested [9, 31] and the population used for developing the model (GWAS population). PRSs are built upon our existing comprehension of genetic associations, which is far from complete. Numerous genetic factors might not have been identified or incorporated into PRS calculations, indicating the inherent gaps in our knowledge (see below for a discussion of intermediate steps between genotype and phenotype).

Moreover, PRS can exhibit ethnic biases since many are formulated using genetic data from specific populations, potentially introducing biases and inaccuracies when applied to individuals from different ethnic backgrounds.

Importantly, current PRS methodologies do not account for environmental factors, lifestyle choices and gene–environment interactions, which are pivotal in determining disease risk.

## Complexity and confounding factors in biological systems challenge predictive polygenic risk scoring analyses

**Figure 3:**
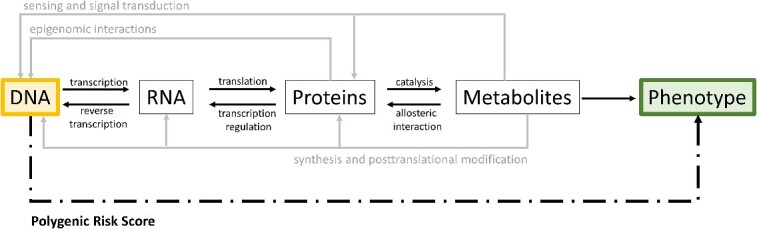
Conventional central dogma of molecular biology, embedded in the more comprehensive ‘modern’ dogma (figure adapted from [38, 51]), which includes potential feedback loops leading to ‘forward’ and ‘reverse’ flow of information. With the concept of PRS, we aim to predict the phenotype directly from the genomic (DNA) information. Thus, in case they are not fully genetically determined, significant influencing interactions are not captured adequately.

Following the central dogma of molecular biology, PRSs link the genetic information to the highest level (for an individual), the phenotype (Fig. 3). Thus, many steps, such as the transcription, protein and metabolite levels, are ‘side-stepped’ as are the many forward, horizontal, and backward regulatory interactions [38–41]. This unresolved complexity leads to a lower power of GWAS, PRS and GP analysis, because many relevant interactions and sources of variability are not considered (for more information, see [42]). This also includes confounding factors hidden in the data and affecting organismic properties [43, 44]. Furthermore, the data underlying the original prediction model may change over time, known as ‘concept drift’. Concept drift analysis, as explained in reference [43], is an underappreciated concept thus far [44]. Minimally, continually checking and/or revising calculated PRS values, by taking into account factors such as the passage of time, to accurately account for the presence of confounding variables, is an essential part of PRS applications.

PRS analysis relies on static DNA information. In contrast, biological systems are dynamic with millions of regulatory interactions aross all levels of molecular organization, as depicted in Fig. 3 [39, 41]. Recently, we provided a systematic analysis of how much phenotypic variance is captured by classical GWAS analysis [42]. Using an in-depth literature survey, we concluded that in the best cases, only 10–50% of variance is captured by genetic information alone. This dynamic interplay between different organizational levels holds significant importance in determining prediction outcomes. Presently, the scientific community is actively addressing this challenge through the field of integrating multi-omics/panomics sciences with GWAS, GP, machine learning, AI and metabolic modelling [42]. To address this problem we have integrated metabolomics, GWAS, GP and data-driven inverse modelling recently in an analysis of 241 *Arabidopsis thaliana* genotypes [45]. This revealed a significant congruence of control points in the metabolomics data by metabolic GWAS and inverse data-driven metabolic models indicating causal relationships from the genotype to the metabotype [45]. In another study, we have applied this metabolomics-driven inverse modelling approach in a multiomics data set to reveal control points of macrophage metabolism [46]. In this study, a checkpoint of macrophage M1/M2 polarization was identified and later confirmed as an immunosuppressive anti-tumorigen switch revealing causal metabolic pathways from genes to molecular phenotypes [46, 47]. In the future we are aiming for the further integration of these principles of data-driven multiomics modelling [40] with GP and PRS and combine it with machine and deep learning strategies to reveal causal pathways from the genome to the phenotype. The ‘modern’ central molecular biology dogma (Fig. 3) [48–50] captures the potential impact of dynamic information flow in forward, backward direction and within a given level of molecular organization. Using PRS, the aim is to predict the phenotype directly from the genomic information (Fig. 3). According to our discussion departures from perfect predictability may then be interpreted as additional contributions of the complex interactions at the dynamic intermediate levels and, in particular, of the environment [41, 42].

## A brief historical overview of GRS and PRS concepts

Over the past few decades, the field of genetics has undergone significant advances in understanding the complex relationship between genetic factors and human traits or diseases. GRS emerged as an early approach, utilizing a limited number of genetic markers with strong associations to predict an individual’s risk for certain single-gene disorders [52]. However, GRS had limited applicability to complex traits influenced by multiple genetic variants. With the advent of GWAS around the mid-2000s, the focus shifted towards studying the entire genome and identifying genetic variants associated with various complex traits [53, 54]. This led to the development of PRS, which aggregates the effects of multiple genetic variants to predict an individual’s overall genetic risk for complex traits or diseases. Since then, PRS has gained substantial traction, as researchers continue to refine methodologies, integrate more genetic data and optimize prediction models.

Another important conceptual development of PRS has been that of including the effects of LD [55]. LD describes the non-random association of alleles at neighboring genetic loci, i.e., the correlation of alleles of neighboring SNPs. Because of linkage, neighboring SNPs often carry redundant information and may lead to inflated PRSs as effects that are truly only associated with a single SNP are spread over all linked SNPs. ‘LD score regression’ has been introduced to better factor in linkage in the estimation of effect sizes and overall heritability of a trait or disease [56]. LD score regression has become a crucial tool for calculating PRS, enabling researchers to weigh the contributions of individual genetic variants more accurately [57]. By accounting for LD patterns, LD score regression enhances the precision of PRS, making it a more robust and effective approach for predicting an individual’s genetic risk for multifactorial traits or diseases. The relationship between LD and PRS underlines the importance of understandingthe genetic basis of complex traits and their potential applications in personalized medicine and risk assessment.

In the following, we will first categorize methods for calculating GRS and PRS and briefly highlight the state of the art in each category. Then, a chronological assessment of tools for computing RS, GRS, and PRS will be provided. These tools are classified by methodology, delineating between RS, GRS or PRS calculations. Furthermore, they are categorized from a user-oriented viewpoint, taking into account factors like operating system compatibility and the scope of calculation capabilities, be it specific or universal.

## Classification of methods for calculating PRS

The classification of methods for calculating PRS encompasses diverse approaches tailored to extract meaningful insights from genetic data. Extending the classification given above [9], we propose to distinguish between the following:

1) Clumping and Thresholding techniques—streamline data by focusing on significant genetic variants and establishing thresholds for inclusion.
Tools belonging to this category: Clinotator [65], PRSice [12], PRSice-2 [68, 69].
2) Genetic correlations and their linked functional annotations—address the interconnectedness of genetic variants and their functional implications, providing a nuanced understanding of genetic risk.
Tools belonging to this category: CanRisk [75], GenRisk [79], JASS [72], impute.me [73], Neptune, PRScs, SumHer [59].
3) Regression-Based Methods—employ statistical regression models to quantify the cumulative effect of genetic variants on disease risk.
Tools belonging to this category: AFA-Recur [82], CanRisk [75], GDM [77], SCFA [74], PXS [66].
4) Bayesian Methods—leverage Bayesian statistical frameworks to infer probabilities and uncertainties in polygenic risk assessment.
Tools belonging to this category: SNP2TFBS [64], SBayesR [70].
5) Other Machine Learning and Optimization Algorithms—such as Support Vector Machines or Genetic Programming [58] utilize sophisticated algorithms to discern complex patterns in genetic data, further enhancing risk prediction capabilities.
Tools belonging to this category: Clinotator [65], CluStrat [71], FCS [66], GenRisk [79], JASS [72], LDPred-2 [63], Neptune, PRScs [67], PUMAS [26], PXS [66], TrumpetPlots [83].

Some tools may appear in multiple categories based on their functionality or classification. Each method contributes uniquely to the refinement and accuracy of PRS calculations, advancing the field of personalized medicine and genetic risk assessment.

## Survey of available GRS/PRS tools

**Figure 4:**
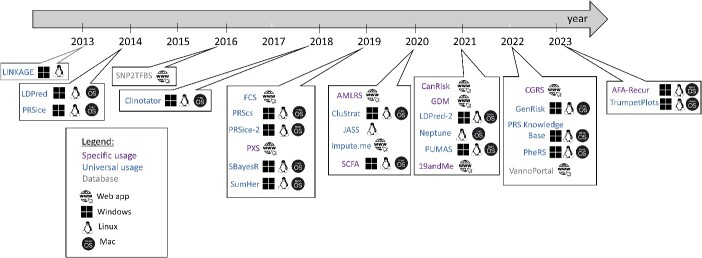
Evolution of tools for calculating RS, GRS and PRS. This figure presents a chronological ordering of available tools for calculating RS, GRS and PRS within their initial decade of development. The tools are categorized based on their methodology, distinguishing between the calculation of RS, GRS or PRS. Additionally, they are divided from a user's perspective, considering factors such as the operating system and the specific or universal calculation capabilities of each tool.

This chapter offers a chronological evaluation of tools designed for computing RS, GRS, and PRS, tracking their evolution over the initial decade of development. These tools are organized based on their methodologies, distinguishing between RS, GRS or PRS calculations. Additionally, they are categorized from a user-centric perspective, considering factors such as operating system compatibility and the breadth of calculation capabilities, whether specific or universal; see Fig. 4 and Table 1. These tools were selected based on functionality, publication or availability on bio.tools website. The other 149 tools that are available in public repositories, such as github or bitbucket web-server, and deal with PRS, are included in the Supplemental Table S1.

**Table 1:** List of tools: column of classification represented division according to the available specificity application scenario of use of the tools (Specific|Universal): this column represents the division according to the potential scope of application scenarios of the tools.

**Name**	**Classification**	**Type**	**License**	**Year**	**URL**	**DOI**	**Maintained**
AFA-Recur	Specific	RS	not specified	2023	http://afarec.hpc4ai.unito.it/	https://doi.org/10.1093/europace/euac145	No
AMLRS	Specific	RS	not specified	2020	https://tcgi.shinyapps.io/amlrs_nomogram/	–	No
CanRisk	Specific	PRS	not specified	2021	http://CanRisk.org	https://doi.org/10.1158/1055-9965.EPI-20-1319	Yes
CGRS	Specific	GRS	CC BY 4.0	2022	http://39.100.117.92/CGRS/	https://doi.org/10.21203/rs.3.rs-74747/v1	No
Clinotator	Universal	RS	GPL-3.0	2018	https://github.com/rbutleriii/Clinotator	https://doi.org/10.12688/f1000research.14470.2	Yes
CluStrat	Universal	PRS	GPL-3.0	2020	https://github.com/aritra90/CluStrat	https://doi.org/10.1101/2020.01.15.908228	Yes
FCS	Universal	RS	not specified	2019	http://bioinfo.cnic.es/FCS	https://doi.org/10.1101/805051	Yes
GenRisk	Universal	PRS	MIT	2022	https://github.com/AldisiRana/GenRisk	https://doi.org/10.1093/bioinformatics/btac152	Yes
GDM	Specific	RS	not specified	2021	https://liuhongwei.shinyapps.io/gdm_risk_calculator/	https://doi.org/10.1002/dmrr.3397	Yes
JASS	Universal	PRS	MIT	2020	http://statistical-genetics.pages.pasteur.fr/jass/	https://doi.org/10.1093/nargab/lqaa003	Yes
impute.me	Universal	PRS	not specified	2020	http://Impute.me	https://doi.org/10.3389/fgene.2020.00578	Yes
LDPred	Universal	PRS	MIT	2015	https://github.com/bvilhjal/ldpred	–	No
LDPred-2	Universal	PRS	not specified	2021	https://github.com/privefl/bigsnpr	https://doi.org/10.1093/bioinformatics/btaa1029	Yes
LINKAGE	Universal	GRS	MIT	2013	http://www.jurgott.org/linkage/LinkagePC.html	–	No
Neptune	Universal	PRS	MIT	2021	https://gitlab.com/bcm-hgsc/neptune	–	Yes
PRScs	Universal	PRS	MIT	2019	https://github.com/getian107/PRScs	https://doi.org/10.1038/s41467-019-09718-5	Yes
PRSice	Universal	PRS	GPL-3.0	2015	http://PRSice.net	https://doi.org/10.1093/bioinformatics/btu848	No
PRSice-2	Universal	PRS	GPL-3.0	2019	http://PRSice.net	https://doi.org/10.1093/gigascience/giz082	Yes
PRS Knowledge Base	Universal	PRS	academic and not-for-profit use	2022	https://prs.byu.edu/ https://github.com/kauwelab/PolyRiskScore	–	Yes
PheRS	Universal	RS	GPL-2	2022	https://phers.hugheylab.org	https://doi.org/10.1093/bioinformatics/btac619	Yes
PUMAS	Universal	PRS	MIT	2021	https://github.com/qlu-lab/PUMAS	https://doi.org/10.1186/s13059-021-02479-9	Yes
PXS	Specific	PRS	not specified	2019	http://apps.chiragjpgroup.org/pxs/	https://doi.org/10.1101/833632	Yes
SBayesR	Universal	PRS	not specified	2019	https://cnsgenomics.com/software/gctb/#Download	https://doi.org/10.1038/s41467-019-12653-0	Yes
SCFA	Specific	RS	not specified	2020	https://github.com/duct317/SCFA	https://doi.org/10.3389/fonc.2020.01052	Yes
SNP2TFBS	*Database*		not specified	2017	http://ccg.vital-it.ch/snp2tfbs/	https://doi.org/10.1093/nar/gkw1064	Yes
SumHer	Universal	PRS	GPL-3.0	2019	http://dougspeed.com/sumher/	–	Yes
TrumpetPlots	Universal (visualization tool)	RS	MIT	2023	https://cran.r-project.org/web/packages/TrumpetPlots/index.html	https://doi.org/10.1101/2023.04.21.23288923	Yes
VannoPortal	*Database*		not specified	2017	http://mulinlab.org/vportal	https://doi.org/10.1093/nar/gkab853	Yes
19andMe	Specific	RS	not specified	2021	https://19andme.covid19.mathematica.org	–	Yes

‘Specific’ indicates tools limited to evaluating PRS for a particular purpose and specific disease, while ‘Universal’ denotes tools designed for universal application across various PRS contexts. Type divides tools focus calculation on Risk Score (RS), Genetic Risk Score (GRS) and Polygenic Risk Score (PRS); Year is a year of publication of the tool.

During the early period, until the years 2010–15, foundations of GRS and PRS models were laid. With the advent of state-of-the-art genomics technologies and large-scale genomic data, scientists have expanded the scope of GRS and PRS computations. This led to the futher development of the first GRS and PRS tools described and presented in this chapter.

## The early developments of GRS and PRS

LINKAGE—the first mention of the LINKAGE software tools dates back to 1996 [59]. The LINKAGE comprises a series of programs at its core, serving the purposes of maximum likelihood estimation for recombination rates, lod score table calculations and genetic risk analysis. The last update is from 2013 (see https://www.jurgott.org/linkage/LinkageUser.pdf).

LDPred—LDPred is the predecessor of the LDPred-2 tool [60]. Initially introduced as a method, this tool estimates the posterior mean effect size of each marker by leveraging prior information on effect sizes and LD data [55].

PRSice—the first dedicated PRS software. PRSice [12] (‘precise’) offers a comprehensive suite of functionalities for calculating, applying, evaluating, and visualizing PRS results. PRSice allows PRS calculations at various thresholds, accommodating high-resolution analyses, as well as broader *P*-value thresholds. It handles genotyped and imputed data, incorporates ancestry-informative variables, and can simultaneously apply PRS analysis across multiple traits [12].

SNP2TFBS – computational resources and databases such as SNP2TBS are also crucial for PRS development and calculations. SNP2TFBS serves as a computational resource designed to assist researchers in exploring the molecular mechanisms involved in regulatory variation within the human genome [61].

Clinotator—the tool takes input variants and utilizes NCBI E-utilities to produce ClinVar Variation Report scoring metrics [62]. Its primary objective is to generate annotations relevant to batches of variants to aid in clinical interpretation. The scoring metrics include Clinotator Raw Score, Average Clinical Assertion Age, Clinotator Predicted Significance and classification Recommendation [62].

## State-of-the-art GRS and PRS tools

FCS—FCS Frequency Conservation Score for detecting pathogenic single nucleotide variants in nuclear and mitochondrial DNA [63]. These scores are based on a random forest model trained using various predictors, locus variability from the gnomAD database, and physicochemical distance for amino acid substitutions and impact over the canonical transcript.

PRScs—PRScs uses a high-dimensional Bayesian regression framework. This unique approach exhibits robustness across diverse genetic architectures, delivers significant computational benefits, and facilitates multivariate modeling of local LD patterns [64].

PRSice-2—PRSice-2 is an upgraded version of PRSice, which offers significantly improved speed and memory efficiency compared with PRSice-1, LDpred and lassosum while maintaining comparable predictive performance [65, 66].

PXS—PXS is a web tool that upgraded PRS into Poly-exposure score (PXS). It is based on an additive modelling approach to estimate and validate a PXS that extends beyond considering a limited number of factors, such as smoking and pollution [63].

SBayesR—the SBayesR tool extends a robust individual-level data Bayesian multiple regression model called BayesR to leverage summary statistics from GWAS [67]. SBayesR enhances prediction accuracy compared with commonly employed state-of-the-art summary statistics methods, all while consuming a significantly lower amount of computational resources [67].

SumHer—SumHer [56] is a tool for a summary of statistical analysis. It is presented as an auxiliary tool to improve the PRS calculation.

AMLRS—a web-based prognostic tool to forecast the prognosis of Acute Myeloid Leukemia (AML) in patients. By employing a log-rank test, univariate COX regression analysis and LASSO-COX, it identified 10 survival-related genes that constituted the AML Risk Score [68].

CluStrat—the CluStrat tool [68] introduces a structure-informed clustering approach for population stratification. The method leverages GWAS data to estimate the effects of trait-associated alleles and calculate PRS, providing valuable insights into the genetic architecture of the studied traits.

JASS—the software package JASS [69] efficiently computes joint statistics for selected GWAS results and facilitates interactive exploration of the findings via a user-friendly web interface.

impute.me—the tool impute.me offers advanced DNA analysis that goes beyond individual SNPs, empowering users with comprehensive genetic information [70]. The web-based engine is providing state-of-the-art trait and disease genetic scores based on advanced polygenic risk scoring.

Subtyping via Consensus Factor Analysis (SCFA)—SCFA is a groundbreaking approach for cancer subtyping and risk prediction known as Subtyping via Consensus Factor Analysis (SCFA) [71]. This method effectively eliminates noisy signals, retaining consistent molecular patterns, thereby enabling the reliable identification of cancer subtypes and accurate RS predictions for patients.

CanRisk—CanRisk [72] is an innovative web interface for the Breast and Ovarian Analysis of Disease Incidence and Carrier Estimation Algorithm risk prediction model [73]. It is the first comprehensive model to enable reliable breast cancer risk prediction in unaffected women, common cancer genetic susceptibility variants using PRS, explicit family history, personal lifestyle, hormonal and reproductive risk factors, and mammographic density.

Gestational diabetes mellitus (GDM) —this tool uses a machine learning-based prediction model specifically tailored for Chinese women in early pregnancy to accurately predict the likelihood of GDM [74].

Neptune—Neptune (https://gitlab.com/bcm-hgsc/neptune) is an innovative system designed to facilitate seamless interaction between a clinical laboratory and an electronic health record system, creating an environment for delivering genomic medicine with immense potential for enhancing healthcare. This tool required customizable clinical reports encompassing various genetic data types, such as SNVs, CNVs, pharmacogenomics and PRS.

LDPred-2—LDpred2 [60] is an updated version of LDpred designed to calculate PRS. It introduces two new options: a *'sparse'* option capable of learning effects that equal zero and an *'auto'* option that directly learns the two LDpred parameters from the data. In benchmark tests using simulated and real data, LDpred2 outperforms its predecessor LDpred1, showcasing enhanced robustness and predictive accuracy.

PUMAS—PUMAS (A Novel Method for Fine-Tuning PRS Models Using GWAS Summary Statistics) [24] offers a cutting-edge approach to fine-tuning PRS models using summary statistics from GWASs.

19andMe— ‘19 and Me: COVID-19 Risk Score Calculator’ is an innovative tool that combines reported COVID-19 geographic case data and up-to-date scientific research to estimate the potential risk the disease poses to an individual.

Clinic and Genetic Risk Score (CGRS)—the CGRS calculator is a web application designed to assess the prognosis of gastric cancer patients [75].

GenRisk—GenRisk is a Python package that leverages various gene-based scoring schemes to analyze and identify significant genes associated with a phenotype in a population [76]. It enables the computation and integration of gene scores, considering both rare deleterious variants’ burden and common-variants-based PRS.

PRS Knowledge Base—PRS Knowledge Base serves as a centralized online repository, enabling users to calculate and contextualize PRS (https://github.com/kauwelab/PolyRiskScore).

PheRS—the PheRS tool [77] calculates PRS derived from electronic health records to investigate Mendelian diseases and rare genetic variants. The phers R package was developed to address this as a comprehensive and user-friendly collection of functions and maps that facilitate a PheRS-based analysis of linked clinical and genetic data [77].

VannoPortal—VannoPortal web is a comprehensive variant annotation database, consolidating and integrating genome-wide variant annotations and prediction scores from diverse biological domains [78]. These domains include allele frequency, LD, evolutionary signature, trait association, pathogenesis, allele imbalance, base-wise functional prediction, and tissue- or cell-type-specific functional profiles.

AFA-Recur—AFA-Recur, a machine-learning-based probability score, demonstrates predictive performance in estimating the 1-year risk of recurrent atrial arrhythmia following AF ablation [79]. This freely accessible online calculator offers patient-specific predictions, enabling tailored therapeutic approaches for individual patients.

TrumpetPlots—the tool visually represents the association between an allele frequency and effect size in genetic studies [80]. It takes as input a data frame comprising association results and generates a plot that displays the effect size of risk variants on the Y-axis and the allele frequency spectrum on the X-axis.

## Summary

The concept of the PRS emerged at the forefront of genetic research around the year 2008, with reproducible software tools starting to be published in subsequent years, particularly around the year 2018. It represents a significant advancement in capturing the genetic basis of complex traits and diseases. Implementing the PRS involves utilizing various computational tools and methodologies to calculate and aggregate the effects of multiple genetic variants associated with a specific trait.

Researchers continuously work to optimize and refine the PRS methodology by exploring novel approaches and incorporating the latest advances in genomics and statistical methods. As a powerful predictive tool, the PRS holds immense promise for personalized medicine and risk assessment in various fields, from healthcare to behavioural genetics, as was already proven in the first decade of GRS and PRS development.

## Future outlook

The future of GRS and PRS shows immense potential in support of precision genetics and personalized healthcare. With ongoing technological advancements, we anticipate significant progress in data collection, genomics and computational methodologies, enabling the incorporation of even more genetic variants into GRS and PRS calculations. These refined models will offer improved accuracy and enhanced predictive power, enhancing risk assessment for a wide array of complex traits and diseases. Moreover, continuous research could deepen our understanding of the intricate interplay between genetics and environmental factors.

While PRSs exhibit strong reproducibility, they account for only a fraction of the genetic variance and lack the inclusion of interactions. A more realistic scenario suggests the existence of numerous independent marginal effects alongside a vast array of interaction effects. Current research offers extensions to the PRS methodology [81, 82] to address these problematic effects, and showcases a notable role of gene–gene interactions in bipolar disorder.

By integrating environmental data, lifestyle information, and other omics data (e.g., epigenomics, metabolomics) into GRS and PRS models, we can adopt a comprehensive and holistic approach to individual risk assessment. This will pave the way for personalized interventions and targeted prevention strategies. A primary focus in the future will be the integration of GRS and PRS into clinical practice, allowing healthcare providers to tailor treatment plans based on each patient’s unique genetic risk profile.

While PRS tools are still in their early stages of development, they hold the potential to revolutionize disease screening and early detection, ultimately leading to more tailored and effective healthcare strategies. Notably, there are also promising applications in plant cultivation, with studies beginning to emerge in this field [83].

**Key Points**

- Genetic Risk Scores (GRS) and Polygenic Risk Scores (PRS) hold great potential for precision medicine predictive analyses.
- Advancements in data collection, genomics and computational methodologies are expected to enable the inclusion of a larger number of genetic variants in GRS and PRS calculations.
- This survey provides a brief overview of GRS and PRS tool developments.
- Integrating environmental data, lifestyle information and other omics data into GRS and PRS models will provide a more comprehensive approach to individual risk assessment, enabling personalized interventions and targeted prevention strategies.

## Supplementary Material

Supplementary_Table_S1_bbae240

## Author Biographies

**Jana Schwarzerova** is a PhD candidate in the Bioinformatics research group in the Department of Biomedical Engineering of the Brno University of Technology. Her main research area is inverse and reverse modelling in systems biology, and omics analysis using the bioinformatics approach.

**Martin Hurta** is a PhD student in the Evolvable Hardware research group in the Department of Computer Systems at the Brno University of Technology. His main research is genetic programming and its use for the design of hardware accelerators.

**Vojtech Barton** is a PhD candidate in the Bioinformatics research group in the Department of Biomedical Engineering of the Brno University of Technology. His main research area is in bioinformatics.

**Matej Lexa** is an associate professor at the Faculty of Informatics, Masaryk University. His field of research is bioinformatics.

**Dirk Walther** is a Bioinformatics group leader at the Max Planck Institute of Molecular Plant Physiology, and an Adjunct professor at the Potsdam University.

**Valentine Provaznik** is a full professor at Brno University of Technology and head of the Department of Biomedical Engineering of the Brno University of Technology. She is the PhD supervisor of JS.

**Wolfram Weckwerth** is a full professor at University of Vienna, head of Department Functional and evolutionary Ecology and Director of the Vienna Metabolomics Center (VIME), University of Vienna. He is the PhD co-supervisor of JS.
